# A Qualitative Study Investigating the Barriers to the Implementation of the ‘Sepsis Six Care Bundle’ in Maternity Wards

**DOI:** 10.3390/healthcare8040374

**Published:** 2020-10-01

**Authors:** Nouf Abutheraa, Alexander B. Mullen, June Grant, Gazala Akram

**Affiliations:** 1 Strathclyde Institute of Pharmacy and Biomedical Science, University of Strathclyde, 161 Cathedral Street, Glasgow G4 0RE, UK; a.mullen@strath.ac.uk (A.B.M.); gazala.akram@strath.ac.uk (G.A.); 2 NHS Greater Glasgow & Clyde, Princess Royal Maternity, 16 Alexandra Parade, Glasgow G31 2ER, UK; June.grant@ggc.scot.nhs.uk

**Keywords:** sepsis, care bundle, behaviour, implementation science, midwives, sepsis six care bundle

## Abstract

*Background:* In 2014, the Sepsis Six Care Bundle (SSCB) was introduced into a Scottish health region to improve patient outcomes. Poor compliance was demonstrated with the SSCB across different specialities. This study explored determinants of non-compliance with the SSCB in maternity wards. *Methods:* In-depth interviews were conducted with midwives in a single Scottish health region. Convenience sampling was used to recruit interviewees. The interviews were digitally recorded, transcribed verbatim, entered into NVivo software, and analysed using thematic analysis. *Results:* Thirteen face-to-face interviews were completed and lasted an average of 33 min. Three main barriers were identified to SSCB implementation; the difficulty of diagnosing sepsis, the suitability of the SSCB in a maternity setting as part of the pre-conditions phase, and the lack of staff training as part of the pre-implementation phase. *Conclusion:* The findings emphasize the importance of adapting improvement initiatives with sufficient preparation of staff in the rationale use to the context of care bundles.

## 1. Introduction

The World Health Organization’s Global Maternal Sepsis Study (GLOSS) published prospective data collected over a 1-week period across 52 counties and found that confirmed or suspected sepsis had an incidence of 70.4 in every 1000 live births, and about 10.9 in every 1000 women experience a severe maternal outcome. Obstetric infections account for 10.7% of maternal mortalities, primarily due to maternal sepsis [1]. A UK national cohort study found that 14.4% of maternity admissions experienced severe sepsis and needed critical care with 10.6% progressing to septic shock. The absolute risk of critical care admission from severe sepsis in maternal women was 4.1 (95% CI 2.9–5.6) in every 10,000 maternities [2]. In Scotland, an incidence rate ranging from 1.08 to 1.65 cases of sepsis in every 1000 maternities has been reported [3].

An accurate assessment of infection occurring during maternity represents a complex clinical assessment challenge due to the changes in pregnant female physiology and affects 11.9 million women each year. Sepsis is a life-threatening condition resulting in organ dysfunction caused by a dysregulated host response to infection and can progress to death [1,4].

Therefore, the prevention and emphasis on early diagnosis of sepsis is critical to enhance the management of maternal sepsis and has led to several “care bundles” being implemented in general wards [1]. A care bundle is “a small set of evidence-based interventions for a defined patient segment/population and care setting that, when implemented together, will result in significantly better outcomes than when implemented individually” [5]. Care bundles have been introduced into clinical practice to avoid unnecessary delays in the delivery of care and to encourage the adoption and acceptance of evidence-based practice [6]. The Sepsis Six Care bundle (SSCB) had an extended implementation in maternity wards to improve clinical monitoring and to reduce the mortality and morbidity of women giving birth. [7] It was introduced into a Scottish Health Board region in 2014 (Appendix A) for implementation whenever sepsis was suspected and supported by two abnormal systematic inflammatory response syndrome (SIRS) values. The SIRS was considered to be met if at least two of the following were present: temperature, heart rate (HR), white cell count (WCC) or respiratory rate (RR) [4].The SSCB was used to document the care that was or was not delivered, and to further support communication within the healthcare team, a sepsis six “sticker” is attached to the patients’ medical (paper) notes. The purpose of the sticker was to alert staff and to promote the delivery of all six elements of the bundle within one hour of sepsis being diagnosed.

Over 70 strategies and frameworks have been evaluated to improve the uptake of care bundles [8,9]. The Replicating Effective Programs Framework, for example, has four phases: preconditions, pre-implementation, implementation, and maintenance and evolution [8,10]. The precondition phase aims to identify barriers, needs, and effectiveness for new interventions and its fit within local settings [8,10]. The pre-implementation phase includes the development of working groups and staff training, which is followed by an implementation phase that includes assessment of technical issues, feedback, and consideration of any improvements. Finally, the maintenance and evolution phase covers the need to revise the programme after delivery [8,10].

Nurses and midwives have been shown to have key roles in the effective implementation of sepsis bundles [11]; however, an assessment of the use of the SSCB in maternity wards found low rates of SSCB implementation [12]. The aim of the current study was to explore the barriers to the implementation of the SSCB in maternity wards in a single Scottish Health Board area.

## 2. Materials and Methods

In-depth interviews were conducted with midwives in a single Scottish health region by approaching each maternity ward and asking the staff available about their interest in participating in the study. Research interviews were only conducted between 0900 and 1700 due to the availability of a single researcher to conduct the research.

### 2.1. The Quality Criterion

The Consolidated Criteria checklist for Reporting qualitative research (COREQ) was used to ensure all aspects of the process were appropriately reported [13]. A full list of the details in the relevant data is available as supplementary material S1.

- **Research team and reflexivity**
  The interviews were conducted by the main author (N.A.), a female doctorate student who was experienced with conducting and analysing interviews. The interviewer had previously undertaken an observational study in the same ward [12] and had become familiar with ward staff and gained the trust of the midwives who were aware of the reason for her presence and subsequent interviews.
- **Study design**
  The study methodological orientation was based on grounded theory [14] convenience sampling was used to recruit interviewees who were working between 0900 and 1700 when the interviewer was present in the ward. Sampling was conducted over a seven-week period with a target to include a minimum of one midwife from each maternity ward. Face-to-face interviews were conducted with 13 midwives; data on interviewees’ clinical experience within maternity wards was collected. Interviews ceased once data saturation was achieved, in other words, when similarities of information appeared with no new details emerging from the interviews. All interviews were conducted in a quiet area of either the maternity ward or the High Dependency Unit (HDU), depending on each participant’s area of practice.
- **Data analysis**
  Interviews were audio recorded, transcribed verbatim, and checked for accuracy, although “field notes” had not been taken during any interviews. The interviewees did not review the transcripts due to time and logistical issues. The data were analysed using a thematic analysis approach with the help of NVivo V11 software (QSR International Pty Ltd., Doncaster, Victoria, Australia). The data were independently verified by G.A., who reviewed two of the original transcripts and consensus around emerging codes and themes was obtained.

### 2.2. The Interview Guide

An interview guide (Appendix B) was developed and validated by the clinical risk manager at one maternity hospital, by a qualitative research associate within the institute, and by all co-authors (G.A., A.B.M., and J.G.) who had either academic or clinical experience of conducting and supervising qualitative research. The interview guide was piloted with one senior midwife and required no further changes or amendments.

The interview guide intended to explore interviewees’ experiences, opinions, and knowledge in the use of the SSCB. An in-depth interviewing technique was used as it is a robust method to provide “rich data”. Unlike semi-structured or structured interviews, it does not provide a narrative of a theory or collect participants’ experiences in specific aspects but, rather, opens the discussion and allows participants to lead the way in the interview. This method was selected to reduce any preconceptions or bias and allow the topic of interest to be widely explored.

### 2.3. Ethics and Consent

The study protocol was reviewed by the University of Strathclyde Ethics Committee and approval was obtained prior to data collection (Reference # 0001.2016). The scientific officer from the West of Scotland Research Ethics Service also reviewed the study design and advised that it was a service evaluation and therefore did not require a full ethics submission. The study design was approved by the local NHS Caldicott Guardian and was compliant with national data governance policies.

A consent form was used with all participants (Supplementary S2). Before being interviewed, each participant was required to sign and date the form after reading the invitation letter and participants’ information sheet (Supplementary S3 & S4).

## 3. Results

A total of 13 midwives from three different sites, each from a different ward, were interviewed. Interviews lasted an average duration of 33 min (range 17.7–62 min). All participants consented and nobody withdrew consent. Participants had an average of 15 years of experience within maternity wards and were working either in postnatal/antenatal wards or an HDU/labour ward. Three main themes were identified from the interview data. These were; difficulty of diagnosing sepsis, the suitability of the SSCB in a maternity setting as part of the pre-conditions phase, and the lack of staff training as part of the pre-implementation phase.

### 3.1. Sepsis: A Difficult Diagnosis

The challenge of identifying sepsis cases was found to be related to the less specific and sensitive SIRS score. If a patient had two abnormal values, she would be started on the SSCB. Pyrexia in labour, for example, could cause medical staff to initiate the SSCB:

*“Somebody who commences Sepsis Six for maternal temperature in labour while their body is doing a lot of work, I expect their temperature to be raised. So, I think there’s far too many women who are commenced on Sepsis Six but they don’t have to be, and a lot of babies get antibiotic cover as well.”*

Participant 4, postnatal ward, >10 years experience.

Interviewees stated that the application of their experience in assessing suitability was fundamental. The reliance on the SSCB could have resulted in some patients receiving unnecessary treatment.

*“Certainly, we don’t have a problem reviewing people, but sometimes when you jump in too quickly it can be. somebody’s due paracetamol at that point. It’s a whole variety of... When sick people come in you know that they’re sick. If they are walking in smiling, then you know that they are not septic.”*

Participant 10, maternity assessment unit, >10 years experience.

Interviewees acknowledged that a diagnosis of suspected sepsis could result in the inappropriate use of antibiotics if the diagnosis was ultimately disproved.

*“... We should not be giving people too much antibiotics. That causes its own problems .... But that’s what you have to (do), act on the side of caution, because sepsis is such a serious thing.”*

Participant 9, postnatal ward, <10 years experience.

Other interviewees acknowledged their worries about the woman’s condition deteriorating, as this could have them commenced on intravenous (IV) antibiotic treatment very quickly to prevent poor clinical outcomes.

*“With it being so important ... I would rather be treated when it’s needed treated, to save anything from getting worse.”*

Participant 13, postnatal ward, >10 years experience.

Community midwives could refer postnatal women to the hospital when they have a slightly elevated temperature. This concern results in suspecting and treating women for sepsis based on pyrexia and a slightly elevated pulse.

*“I think we overprescribe antibiotics, so we get a lot of people in- just caution ... Now when these women are coming in and we are treating them because the community midwives will go out, check their temperature, the temperature setting at 37.6 or something, their pulse is slightly raised. That’s a normal physiological thing during breast feeding. All these women are coming in getting IV antibiotics, they’re absolutely fine when their inflammatory markers come back. So it is just a matter of experience of knowing ‘is that breast milk or is it sepsis?”*

Participant 10, maternity assessment unit, >10 years experience.

### 3.2. Pre-conditions Phase: Suitability of the SSCB in a Maternity Setting

It is important to understand the challege that adoption of both the SSCB and the sepsis protocol brings across the maternity setting. It was noted that there was a strong reliance on the sepsis protocol by some interviewees as they considered the SSCB as a set of initial responses that should be applied indiscriminately to all patients:

*“I think the fact that it’s a set of guidelines for sort of immediate initial management for patients over a one-hour period ... I can kind of get that, that would fit everybody, because it’s almost like an emergency response as a first response and you will do all of these, and then once we’ve got the results of all these investigations, we can then tailor the plan from there.”*

Participant 9, postnatal ward, <10 years experience.

However, some elements within the SSCB were not delivered to patients with suspected sepsis as it was not required. Interviewees treated the SSCB as a list of items that could or could not be commenced to the patient after a medical examination:

*“If ever it’s needed, it’s all there, but you can use it, or the medical staff can use their judgement to say ‘Right. Actually, she does not need that’.”*

Participant 13, postnatal ward, >10 years experience.

For example, the administration of oxygen and insertion of a catheter were not considered relevant in many patients with suspected sepsis, but the administration of intravenous (IV) antibiotic, IV fluid, blood culture, and other blood tests were commenced in many of these patients:

*“I think four out of the six, because we don’t always give oxygen, we don’t always do urine. It just depends.”*

Participant 10, maternity assessment unit, >10 years experience.

If the full SSCB was commenced, there is the possibility of delivering unnecessary care to the patients. Interviewees were concerned that patients could be catheterized or started on IV antibiotics unnecessarily. Despite this belief, they stated that they would not be putting patients in any harm if unnecessary care was delivered to their patients.

*“You wouldn’t get into trouble … for it. They would maybe just get you to read up your protocol again but you would not be doing anybody any harm.”*

Participant 12, HDU/labour ward, <10 years experience.

Interviewees suggested that sepsis is critical, and it might not be ideal for women to be treated for it in a postnatal ward, and, if women are admitted to the postnatal ward, then they do not need the complete SSCB.

*“Somebody in a postnatal ward who requires a Sepsis Six sticker, to me, should not be in the postnatal ward. She should be transferred out of the postnatal ward, if they require all the criteria that is on it.”*

Participant 4, postnatal ward, >10 years experience.

In addition, the ability shown by midwives to discuss the appropriateness of care with colleagues and to involve a senior person in these conversations reflects their strong belief that this care should not be given to every patient diagnosed with sepsis.

*“If it’s a new midwife ..., we’ll say ‘Look, she might not need that. Do you want to discuss that with ... a doctor?’ Or if it’s maybe a junior doctor (and) we felt what happened, in our experience, that we wouldn’t normally do that, we’d say ‘Can you just double check with your registrar?’ ... So we’ll get them to check with somebody more senior”*

Participant 13, postnatal ward, >10 years experience.

Individualized patient care can save patients from being exposed to unjustified treatment and avoid exposing the mother and the baby to unwarranted therapy that can lead to antimicrobial resistance and conflict with the principles of antimicrobial stewardship.

*“Some people will be catheterized unnecessarily or possibly commenced on IV antibiotics unnecessarily. So that’s my concern of having the sticker.”*

Participant 4, postnatal ward, >10 years experience.

Midwives’ practice in these cases will be to complete the requirements of the Sepsis Six protocol, while their feelings and judgment are that the bundle is unnecessary.

*“None of us have a crystal ball that can tell what’s going to happen in the future, so you just have to go with the protocol and (against) your judgment sometimes.”*

Participant 13, postnatal ward, >10 years experience.

### 3.3. Pre-Implementation Phase: Staff Training

There was limited awareness concerning implementation and introduction of the SSCB in maternity wards, particularly in the postnatal/antenatal wards. However, participants from labour wards or the HDU appeared to have been well trained in the use the bundle:

*“If they want to introduce it to the postnatal ward (it’s essential) that they explain it to people before they do, because I was just told ‘Sepsis Six–there is a drawer with packs in it’.”*

Participant 4, postnatal ward, >10 years experience.

In some wards the actual “sticker” was not made available, whilst in other wards the SSCB had been introduced in the form of a “physical pack”, which contained all of the required tools, such as an oxygen mask, catheter, blood bottles, and the sticker.

These findings suggest there might have been a misunderstanding in the management of sepsis cases and the use of the SSCB. Uncertainty of whether they should be giving priority to the patient or the SSCB sticker was observed with participants:

*“Someone who’s unwell, would I go and get the sticker so I could follow the list, or would I wait until everything had been dealt with and then get the sticker, so ‘I did that, I did that’.”*

Participant 9, postnatal ward, <10 years experience.

Some midwives lack senior support and guidance and were unable to visualize the sticker which made them unable to commence the bundle or deliver the care to their patients.

*“I don’t think that I can tell you what the sticker looks like. I’m not sure that anyone has ever shown me the sticker and what to do with it.”*

Participant 9, postnatal ward, <10 years experience.

Some midwives complained of the inconvenience of using a sticker, but preferred the possibility of having a page within the patients’ notes, to which they could turn whenever needed.

*“I suppose with it being a sticker, then you have to go and get it, and stick it in. Whether this is the best thing, or should it be something somewhere in the notes, a page that you turn to, ‘We are now doing sepsis’.”*

Participant 9, postnatal ward, <10 years experience.

Interviewees were concerned about the absence of the Sepsis Six Care Bundle from the ward and the effect it might have on their practices.

*“We don’t have the sticker so we do not follow it the way that they possibly do.”*

Participant 4, postnatal ward, >10 years experience.

## 4. Discussion

There are several major determinants which influence how the SSCB is used in maternity wards relating to the difficulty of diagnosing sepsis, the suitability of the SSCB in a maternity setting as part of the pre-conditions phase, and the lack of staff training as part of the pre-implementation phase.

The challenge of diagnosing sepsis in maternity care plays an important role as many therapies were initiated for suspected rather than confirmed sepsis. Evidence shows that inaccurate diagnosis is found to be the main barrier to implementing care bundles, while the second most significant barrier is staff knowledge, training, and the provision of staff resources to successfully implement the care bundle [15]. A systematic review of seven clinical studies found that the implementation of the sepsis guidelines failed to enhance the delivery of care to sepsis patients. Instead, patients with suspected sepsis were over-treated when they were actually well and needed no therapy [15]. This might be a result of the use of the nonspecific SIRS criteria as these values can be elevated in cases of non-infectious conditions, such as burns or injury [16]. Furthermore, the alterations in female physiological function that occur during pregnancy must be considered when managing patients with sepsis [17].

The SSCB aims to deliver six elements of care to every patient within one hour of sepsis being suspected [18]. However, in this study, we found that women treated for sepsis, especially in a ward setting, were not subject to the complete application of the care bundle. Their oxygen saturation levels were good, which disqualified them from receiving oxygen therapy, and they were not routinely catheterized. The new definition of sepsis as a “life-threatening organ dysfunction caused by a dysregulated host response to infection” [19] is favourable to this finding, as those women who were treated for sepsis in the ward did not experience a life-threatening organ dysfunction; therefore, neither oxygen nor a catheter were needed. It is important to distinguish in the diagnosis between infection, sepsis, and sepsis-related organ dysfunction, as the diagnosis will influence the care delivered to each patient.

Precondition is defined as a “factor that is necessary in order for an implementation mechanism to be activated” [20]. During this period, attention to the intervention is carefully considered to allow the implementation strategy to succeed and for its outcome to occur [20,21]. A cardiovascular (CV) toolkit aimed at reducing CV risk in primary care considered a pre-conditions phase in its implementation. It required the researcher to have a focus group of primary care providers and patients who were able to identify barriers and facilitators to effectively implement the CV risk management tool [18]. This was missing with the SSCB as midwives were not involved in any element of the precondition implementation phase. Midwives only heard about the SSCB during routine practice or found it documented in a patient’s notes. The literature shows [21,22] that most of the implementation strategies are in the precondition phase, which indicating the importance of the early planning for implementation studies.

These findings indicate an inadequate strategy in the education of staff in preparing them for the implementation of the new bundle. It also highlighted that midwives working in HDU/labour wards had a better introduction to the care bundle, improving their awareness over other midwives practicing in postnatal/antenatal wards. It is known that the education aspect of any implementation process is complex and includes five stages: planning, analysis, design, implementation, and development. Some education/training programmes have been found to have no effect on patients’ care [23,24]. A meta-analysis found a correlation of 0.04 between work performance and training outcomes and 0.29 between work performance and effective training reaction [23]. Furthermore, evidence suggests that the lack of senior support and inadequate environmental settings can hinder the education model [24]. This could be improved by the involvement of staff in the design and implementation of the training programme. This was found to be one of the drivers to empower education adoption and overcome the challenge of ineffective education management [24]. Insufficient training has previously been identified as a barrier to implementing care bundle in general hospital settings, along with insufficient resources, poor communication, and a lack of auditing and feedback [25]. In addition, some studies indicate that the education and involvement of the multidisciplinary teams (MDTs) enhances compliance with care bundles [26]. Education programmers, when associated with a curriculum, have the potential to shape healthcare knowledge and skills [27]. For these reasons, more formal education around the SSCB should be considered as part of midwives’ continuous professional development. The curriculum of the training could be developed in collaboration with the MDTs and could also include a refresher course or multi-level training.

Physicians’ behaviours were not investigated as part of this study. However, the literature shows that physicians were motivated to overprescribe for fear of litigation due to a delay in initiating antibiotic treatment. In this study, midwives were aware of the global problem of antibiotic resistance and recognized the need to reduce the use of broad-spectrum antibiotics, but they also resisted going ”too narrow” and inadequately treating a potential infection [28]. The fear of negative patient outcomes from delaying antimicrobial treatment of a potential infection influenced physicians to more readily prescribe. Physicians reported experiencing pressure to prescribe even if the patient was clinically stable and this was in contrast to little stigma or rebuke if patients developed antimicrobial resistance or a *Clostridium* difficile infection resulting from the overuse of antimicrobials [28]. The findings of this study confirm that antimicrobial therapy was frequently prescribed as a “precaution” to prevent patients from deteriorating.

This study was limited to the experience of a single health board (region) and possibly only reflects the practice of healthcare professionals within this setting. A wider evaluation and assessment of different maternity units is worth exploring. This study also acknowledges a limitation in the recruitment process, as the participants in some wards were selected by senior staff, which challenges the interview process as there was some observed hesitation from participants wanting to avoid any conflict with the senior staff. Finally, conducting the interviews in the ward, albeit a quite area, may have also affected participants’ ability to speak freely which poses another limitation to the findings.

## 5. Conclusions

The finding suggests the importance of appropriate activities for introducing this new initiative in the context of maternity care and for its adaptation to the unique challenges in that care environment. The results also highlight the importance of preparing staff appropriately, for example, training to promote greater understanding of the new initiative and to methods for its implementation and evaluation.

## Figures and Tables

**Figure A1:**
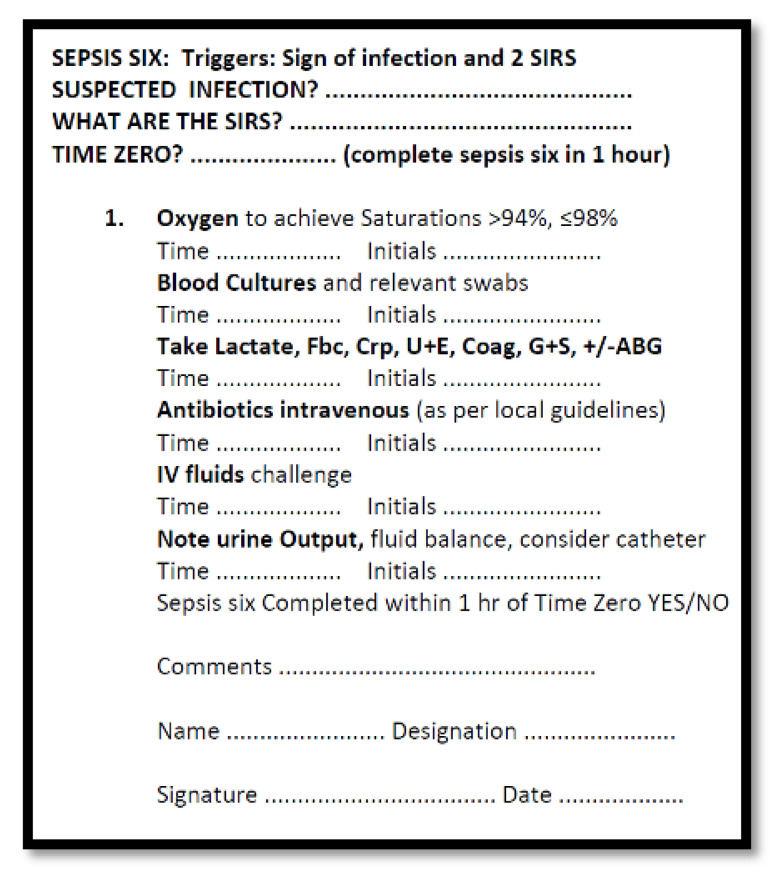
The SSCB sticker that is used in health settings.

## Supplementary Materials

The following are available online at https://www.mdpi.com/2227-9032/8/4/374/s1, Document S1: the COREQ checklist; Document S2: Consent Form; Document S3: Participants’ Information Sheet; Document S4: Participants’ Invitation Letter.

## Appendix B

**Interview Schedule**

**Q1:** What is your occupation? How many years of experience do you have in the NHS in general and in maternity wards?

**Q2:** Before the Sepsis Six Care bundle, how did you assess your patients for sepsis?

**Q3:** Before or after the Sepsis Six Care bundle was introduced, how did your ward prepare you (as a staff member) for using this new tool?

**Q4:** What are your feelings about using the tool now?

**Q5:** Tell me in your own words what is your understanding of the sepsis six care bundle?

❖ How can you identify a patient with sepsis in your ward?
❖ Elaborate on each element in the care bundle
❖ Elaborate on antibiotic de-escalation

**Q6:** How do you continue monitoring your patients after initiating the care bundle?

**Q7:** Many people in your team are involved in delivering the care bundle, but whose responsibility do you think it is to commence the sepsis six care bundle?

**Q8:** As for handover communication, how do you perform the shift handover and the transfer of care handover?

**Q9:** So tell me, before a patient leaves your ward on any normal day, what is the procedure that you follow with every patient regarding their medication?

**Q10:** Are there any other points you want to add?

Thank you for your time and for your valuable participation in this study.
