# Supplementary material for: A Qualitative Study Investigating the Barriers to the Implementation of the ‘Sepsis Six Care Bundle’ in Maternity Wards
Source: Healthcare (Basel). 2020 Oct 1;8(4):374. doi: 10.3390/healthcare8040374 (PMC7712055; doi:10.3390/healthcare8040374)
Supplement: Supplementary file 1 [file healthcare-08-00374-s001.zip › Supplementary File/ S1 The COREQ checklist .docx]

**S1: The COREQ checklist**

|  |  |  |
| --- | --- | --- |
|  |  |  |
| **Domain 1: Research team and reflexivity** | |  |
| **Personal Characteristics** |  |  |
| 1. Interviewer/facilitator | Which author/s conducted the interview or focus group? | All interviews were conducted by one researcher (NA) |
| 2. Credentials | What were the researcher’s credentials? E.g. PhD, MD | MSc Pharmacist |
| 3. Occupation | What was their occupation at the time of the study? | PhD student |
| 4. Gender | Was the researcher male or female? | Female |
| 5. Experience and training | What experience or training did the researcher have? | Attended many course and workshops before conducting the study |
| **Relationship with participants** | |  |
| 6. Relationship established | Was a relationship established prior to study commencement? | The interviewer, by nature of the observational component that was collected previously, had gained familiarity and trust with the maternity staff, who were aware of the reason for the interviews. |
| 7. Participant knowledge of the interviewer | What did the participants know about the researcher? e.g. personal goals, reasons for doing the research |  |
| 8. Interviewer characteristics | What characteristics were reported about the interviewer/facilitator? e.g. Bias, assumptions, reasons and interests in the research topic | Not reported |
| **Domain 2: study design**  **Theoretical framework** | |  |
|  |  |  |
| 9. Methodological orientation and Theory | What methodological orientation was stated to underpin the study? e.g. grounded theory, discourse analysis, ethnography, phenomenology, content analysis | grounded theory |
| **Participant selection** |  |  |
| 10. Sampling | How were participants selected? e.g. purposive, convenience, consecutive, snowball | Convenience sampling |
| 11. Method of approach | How were participants approached? e.g. face-to-face, telephone, mail, email | face-to-face |
| 12. Sample size | How many participants were in the study? | 13 |
| 13. Non-participation | How many people refused to participate or dropped out? Reasons? |  |
| **Setting** |  |  |
| 14. Setting of data collection | Where was the data collected? e.g. home, clinic, workplace | All interviews were conducted in a quiet area of either the maternity ward or the High Dependency Unit (HDU), depending on each participant’s area of practice |
| 15. Presence of non-participants | Was anyone else present besides the participants and researchers? | No |
| 16. Description of sample | What are the important characteristics of the sample? e.g. demographic data, date | Years of experience |
| **Data collection** |  |  |
| 17. Interview guide | Were questions, prompts, guides provided by the authors? Was it pilot tested? | Yes |
| 18. Repeat interviews | Were repeat interviews carried out? If yes, how many? | No - Not reported |
| 19. Audio/visual recording | Did the research use audio or visual recording to collect the data? | audio recorded |
| 20. Field notes | Were field notes made during and/or after the interview or focus group? | No |
| 21. Duration | What was the duration of the interviews or focus group? | An average duration of 33 minutes with a minimum of 17:39 minutes and a maximum of 62 minutes |
| 22. Data saturation | Was data saturation discussed? | Data collection ceased when saturation was achieved, i.e., when similarities appeared with no new details emerging from the interviews. |
| 23. Transcripts returned | Were transcripts returned to participants for comment and/or correction? | No |
| **Domain 3: analysis and findings** | |  |
| **Data analysis** |  |  |
| 24. Number of data coders | How many data coders coded the data? | by one author and validated by another one |
| 25. Description of the coding tree | Did authors provide a description of the coding tree? | Not reported |
| 26. Derivation of themes | Were themes identified in advance or derived from the data? | Drived from the data |
| 27. Software | What software, if applicable, was used to manage the data? | Nvivo |
| 28. Participant checking Reporting | Did participants provide feedback on the findings? | Not reported |
| 29. Quotations presented | Were participant quotations presented to illustrate the themes / findings? Was each quotation identified? e.g. participant number | Yes |
| 30. Data and findings consistent | Was there consistency between the data presented and the findings? | Yes |
| 31. Clarity of major themes | Were major themes clearly presented in the findings? | Yes |
| 32. Clarity of minor themes | Is there a description of diverse cases or discussion of minor themes? | No |
