# Supplementary material for: A Qualitative Study Investigating the Barriers to the Implementation of the ‘Sepsis Six Care Bundle’ in Maternity Wards
Source: Healthcare (Basel). 2020 Oct 1;8(4):374. doi: 10.3390/healthcare8040374 (PMC7712055; doi:10.3390/healthcare8040374)
Supplement: Supplementary file 1 [file healthcare-08-00374-s001.zip › Supplementary File/S2 Consent form 10022017.docx]

**Consent Form**

**Sepsis Six Care Bundle in NHS Greater Glasgow and Clyde Maternity Wards**

| - I confirm that I have read the participant information sheet and fully understand the information provided | □ |
| --- | --- |
| - I confirm that I was given the opportunity to ask questions | □ |
| - I understand that my participation in this study is voluntary and that I am free to withdraw at any time without giving reasons | □ |
| - I understand that the interview will be audio recorded then transcribed | □ |
| - I understand that the data obtained from the interview will be anonymised | □ |
| - I understand that the results may be published | □ |
| - **I agree to take part in the study and participate in the interview** | **□** |

| ___________________ | _ _ / _ _ / _ _ _ _ | __________ |
| --- | --- | --- |
| Name of Participant | Date | Signature |
| ___________________ | _ _ / _ _ / _ _ _ _ | __________ |
| Researcher | Date | Signature |
