# Supplementary material for: A Qualitative Study Investigating the Barriers to the Implementation of the ‘Sepsis Six Care Bundle’ in Maternity Wards
Source: Healthcare (Basel). 2020 Oct 1;8(4):374. doi: 10.3390/healthcare8040374 (PMC7712055; doi:10.3390/healthcare8040374)
Supplement: Supplementary file 1 [file healthcare-08-00374-s001.zip › Supplementary File/S3 Participants Information Sheet .docx]

**Participants’ Information Sheet**

Sepsis Six Care Bundle in NHS Greater Glasgow and Clyde Maternity Wards

**Project background** The Sepsis Six sticker was introduced in 2015 into NHSGGC maternity wards to help ensure that all six items of the Sepsis Six care bundle were delivered within one hour of suspected sepsis. A recent audit conducted in all NHSGGC maternity wards showed low compliance with using the sepsis six sticker. This study is part of a larger study investigating the Sepsis Six Care Bundle and is concerned with determining practitioners’ knowledge of the sepsis six care bundle by exploring their experience and thoughts regarding their use of the sepsis six sticker.

**Who are we recruiting?** Healthcare providers who work in NHSGGC maternity wards and have knowledge of the sepsis six care bundle. It is not crucial that you have used the sepsis six sticker yourself; a basic knowledge of what the sepsis six care bundle is, will be sufficient for you to participate.

**What does taking part involve?** If you decide to take part, a University of Strathclyde PhD student, Nouf Abutheraa (NA), will conduct a short interview with you to explore your knowledge, thoughts and your clinical practice in this area. The interview will take place in a location that is convenient to you and will last no more than 30 minutes. The interview will be audio recorded and then transcribed for analysis.

**Is there is any possible risk?** All data obtained from the interview will be anonymised and handled confidentially. Taking part is completely voluntary. If you agree to take part and then change your mind you are free to withdraw from the study without the need to give a reason.

**What are the possible benefits?** Taking part in this study will help to inform future developments associated with the delivery of care in your ward. It will highlight possible barriers and challenges to the development of sepsis management. The information you provide will be the foundation of developing a quality improvement plan that should provide a better patient experience. The findings may be published and the study will be reported in the researcher’s (Nouf Abutheraa’s) PhD thesis.

**Ethical approval:** This study is sponsored by the University of Strathclyde. An ethics application has been granted by the ethics committee.

**Further questions**: If you require any further information or clarification, please contact the researcher Nouf by email*.*

Nouf Abutheraa, Strathclyde Institute of Pharmacy and Biomedical Sciences University of Strathclyde, 161 Cathedral Street, Glasgow G4 0RE
Email: [nouf.abuhreraa@strath.ac.uk](mailto:nouf.abuhreraa@strath.ac.uk)
