# Supplementary material for: A Qualitative Study Investigating the Barriers to the Implementation of the ‘Sepsis Six Care Bundle’ in Maternity Wards
Source: Healthcare (Basel). 2020 Oct 1;8(4):374. doi: 10.3390/healthcare8040374 (PMC7712055; doi:10.3390/healthcare8040374)
Supplement: Supplementary file 1 [file healthcare-08-00374-s001.zip › Supplementary File/S4 Participants Invitation Letter.docx]

**Sepsis Six Care Bundle in NHS Greater Glasgow and Clyde Maternity Wards**

**Participant’s Letter of Invitation**

You are invited to participate in this study which is investigating the Sepsis Six Care Bundle. It is concerned with determining practitioners’ knowledge of the sepsis six care bundle by exploring their experience and thoughts regarding their use of the sepsis six sticker and you compliance with the care package. This study will help to inform future developments associated with the delivery of care in your ward(s). The aim is to highlight possible barriers and challenges to the development of sepsis management and identify how to better shape care for women who develop sepsis on maternity wards.

**Project background**

The MBRRACE-UK 2009-2012 report states that sepsis accounted for one quarter of maternal deaths in the UK and Ireland. The Sepsis Six sticker was introduced in 2015 on all NHSGGC maternity wards, to help ensure that all six items of the Sepsis Six care bundle were delivered within one hour of sepsis being suspected. A recent audit was conducted in all NHSGGC maternity wards, and its findings showed poor use of the sepsis six sticker, with only one third of patients having received the bundle as part of their hospital care.

This study aims to assess practitioners’ knowledge of the sepsis six care bundle by exploring their experience and thoughts regarding use of the sepsis six sticker.

**Who are we recruiting?**

Healthcare providers who work in NHSGGC maternity wards and who have knowledge of the sepsis six care bundle. It is not conditional that you have used the sepsis six sticker yourself; a basic knowledge of what the sepsis six care bundle is will be sufficient for you to participate.

If you want to particpant, please read the attached participant’s information sheet, and if you require any further information or clarification, please contact the researcher, Nouf Abutheraa (who will conduct the interview) at the Strathclyde Institute of Pharmacy and Biomedical Sciences, University of Strathclyde, Glasgow, [nouf.abuhreraa@strath.ac.uk](mailto:nouf.abuhreraa@strath.ac.uk).You can also contact the academic project supervisor, Professor Alex Mullen of the University of Strathclyde [a.mullen@strath.ac.uk](mailto:a.mullen@strath.ac.uk), or the NHS project supervisor, June Grant of Princess Royal Maternity [June.Grant@ggc.scot.nhs.uk](mailto:June.Grant@ggc.scot.nhs.uk).

**Yours faithfully,**

Nouf Abutheraa
